# Multiple Layers of *CDK5R1* Regulation in Alzheimer’s Disease Implicate Long Non-Coding RNAs

**DOI:** 10.3390/ijms19072022

**Published:** 2018-07-11

**Authors:** Marco Spreafico, Barbara Grillo, Francesco Rusconi, Elena Battaglioli, Marco Venturin

**Affiliations:** 1 Dipartimento di Biotecnologie Mediche e Medicina Traslazionale, Università degli Studi di Milano, Via Fratelli Cervi 93, 20090 Segrate, Italy; marco.spreafico@unimi.it (M.S.); barbara.grillo@unimi.it (B.G.); francesco.rusconi@unimi.it (F.R.); elena.battaglioli@unimi.it (E.B.); 2 Istituto di Neuroscienze, CNR, Via Vanvitelli 32, 20129 Milano, Italy

**Keywords:** *CDK5R1*, lncRNAs, Alzheimer’s disease, miR-15/107, NEAT1, HOTAIR, MALAT1

## Abstract

Cyclin-dependent kinase 5 regulatory subunit 1 (*CDK5R1*) gene encodes for p35, the main activator of Cyclin-dependent kinase 5 (CDK5). The active p35/CDK5 complex is involved in numerous aspects of brain development and function, and its deregulation is closely associated to Alzheimer’s disease (AD) onset and progression. We recently showed that miR-15/107 family can negatively regulate *CDK5R1* expression modifying mRNA stability. Interestingly, miRNAs belonging to miR-15/107 family are downregulated in AD brain while *CDK5R1* is upregulated. Long non-coding RNAs (lncRNAs) are emerging as master regulators of gene expression, including miRNAs, and their dysregulation has been implicated in the pathogenesis of AD. Here, we evaluated the existence of an additional layer of *CDK5R1* expression regulation provided by lncRNAs. In particular, we focused on three lncRNAs potentially regulating *CDK5R1* expression levels, based on existing data: NEAT1, HOTAIR, and MALAT1. We demonstrated that NEAT1 and HOTAIR negatively regulate *CDK5R1* mRNA levels, while MALAT1 has a positive effect. We also showed that all three lncRNAs positively control miR-15/107 family of miRNAs. Moreover, we evaluated the expression of NEAT1, HOTAIR, and MALAT1 in AD and control brain tissues. Interestingly, NEAT1 displayed increased expression levels in temporal cortex and hippocampus of AD patients. Interestingly, we observed a strong positive correlation between *CDK5R1* and NEAT1 expression levels in brain tissues, suggesting a possible neuroprotective role of NEAT1 in AD to compensate for increased *CDK5R1* levels. Overall, our work provides evidence of another level of *CDK5R1* expression regulation mediated by lncRNAs and points to NEAT1 as a biomarker, as well as a potential pharmacological target for AD therapy.

## 1. Introduction

Alzheimer’s Disease (AD) is the most common neurodegenerative disorder, causing a severe and permanent impairment of both cognitive and behavioral functions. It accounts for about 70% of the 50 million people suffering from dementia worldwide and it is currently estimated that, with global population aging, the prevalence of AD will triple by 2050 [1], with a significant economic and social burden on both patients’ families and society.

AD is characterized by a plethora of pathological features, including neuronal loss, dendritic hypotrophy and synaptic alteration, microglial malfunction, cerebrovascular amyloid angiopathy, inflammation, and mitochondrial dysfunction [2,3]. However, the most distinctive features are the presence of extracellular senile plaques, formed by fibrillary β-amyloid (Aβ) and neurofibrillary tangles (NFTs), composed of hyperphosphorylated Tau [4]. Abnormal kinase activity is believed to play a major role in AD pathogenesis [5]. In particular, deregulation of Cyclin-dependent kinase 5 (CDK5), a proline-directed serine/threonine kinase involved in several developmental and physiological processes in the central nervous system (CNS) [6,7], has been suggested to play a pivotal role in the onset of the two main pathological hallmarks of AD by inducing Aβ peptide production and mediating Tau protein hyperphosphorylation [8].

CDK5 requires the p35 regulatory subunit to become active and its kinase activity is strictly dependent on the amount of its activator. p35 is encoded by the cyclin-dependent kinase 5 regulatory subunit 1 (*CDK5R1*) gene, which displays a large and highly conserved 3′-UTR, suggestive of an important role of post-transcriptional regulation in the control of its expression. Indeed, we previously demonstrated that *CDK5R1* expression is regulated at the post-transcriptional level by neuronal ELAV (nELAV) RNA-binding proteins [9,10] and by heterogeneous nuclear ribonucleoproteins A2/B1 (hnRNP A2/B1) [10]. In addition, we recently found that the miR-15/107 family of microRNAs is also involved in negatively regulating *CDK5R1* expression. More interestingly, this group of microRNAs turned out to be downregulated in the hippocampus and cerebral cortex of AD patients while *CDK5R1* mRNA levels were upregulated in AD hippocampus [11].

An additional layer of complexity to the regulation of *CDK5R1* expression that can be relevant for AD pathogenesis might be provided by long non-coding RNAs (lncRNAs). LncRNAs are a highly heterogeneous class of RNA molecules of more than 200 bases in length with no protein-coding capacity. They are involved in the control of gene expression at multiple levels, from nuclear architecture to transcription regulation, mRNA splicing and maturation to mRNA localization and stability, and protein translation and stability to regulation of miRNA activity [12]. Owing to this versatility, lncRNAs are now considered as master regulators of gene expression [13]. In particular, lncRNAs have been shown to post-transcriptionally regulate the levels of several target genes by the formation of lncRNA/miRNA/target gene axes, and the dysregulation of the crosstalk between the two types of ncRNAs has been found to be a crucial contributor to disease pathogenesis [14].

The role of lncRNAs in malignancies and their significance as both diagnostic and prognostic markers has been extensively studied and is well established [15], but an involvement of lncRNAs in the pathogenesis of neurodegenerative diseases is now clearly emerging. In particular, different lncRNAs have been found dysregulated in Alzheimer’s disease and involved in AD pathogenesis by promoting β-amyloid production, including BACE1-AS, 17A, and NDM29 [16]. For example, the expression of BACE1-AS, the antisense transcript of the β-secretase encoding gene *BACE1*, is upregulated in AD brains specimens. BACE1-AS was reported to increase the stability of *BACE1* mRNA and to prevent the binding of miRNA 485-5p, therefore positively regulating BACE1 protein levels and promoting Aβ42 synthesis [16,17].

In the present work, we focused on three different lncRNAs which had the potential for regulating *CDK5R1* expression levels and deserved to be analyzed in AD brain tissues, namely NEAT1, HOTAIR, and MALAT1. NEAT1 (nuclear enriched abundant transcript 1) is a lncRNA that regulates gene expression by binding to the promoter of active chromatin sites [18,19]. Moreover, NEAT1 is known to act as a scaffold for paraspeckles [20], representing specific subnuclear bodies that are involved in gene expression regulation by sequestration and retention of specific RNAs and proteins [21]. Relevantly, NEAT1 levels were found to be deregulated in different neurodegenerative diseases [22]. MALAT1 (metastasis-associated lung adenocarcinoma transcript 1), also known as NEAT2 (nuclear-enriched abundant transcript 2), is predominantly localized to nuclear speckles, where it regulates alternative splicing by modulating the phosphorylation status of SR family of splicing factors [23]. MALAT1 has been linked to several human tumors, in most cases being overexpressed in malignant tissues [24]. Both NEAT1 and MALAT1 have been demonstrated to regulate the expression of members of the miR-15/107 group of miRNAs [25,26], which are known *CDK5R1* negative regulators [11]. HOTAIR (HOX antisense intergenic RNA) is transcribed from the antisense strand of the HOXC locus and represses expression of the downstream HOXD locus together with several genes on other chromosomes. HOTAIR is involved in the control of cell apoptosis, growth, metastasis, angiogenesis, DNA repair and, like MALAT1, it has been shown to be upregulated in different types of cancer [27]. Interestingly, HOTAIR can also serve as a scaffold for Lysine-specific histone demethylase 1A (LSD1) complex and polycomb repressive complex 2 (PRC2) [28]. Since the expression of *CDK5R1* is repressed by LSD1 [29], HOTAIR can potentially impact *CDK5R1* levels.

Here, we demonstrated that NEAT1 and HOTAIR negatively regulate *CDK5R1* mRNA levels, while MALAT1 has a positive effect on *CDK5R1* expression. We also showed that all three lncRNAs positively control the levels of miR-15/107 family of microRNAs. Moreover, we evaluated the expression of NEAT1, HOTAIR, and MALAT1 in AD and control brain tissues. Interestingly, NEAT1 displayed increased expression levels in temporal cortex and hippocampus of AD patients, compared to controls. In addition, we observed a strong positive correlation between *CDK5R1* and NEAT1 expression levels in brain tissues, suggesting a novel molecular marker of AD pathogenesis, warranting further studies. Overall, our work provides evidence of another level of *CDK5R1* expression regulation mediated by long non-coding RNAs, which can also impact on Alzheimer’s disease research.

## 2. Results

### 2.1. NEAT1, HOTAIR, and MALAT1 Long Non-Coding RNAs Differently Regulate CDK5R1 Expression

**Figure 1:**
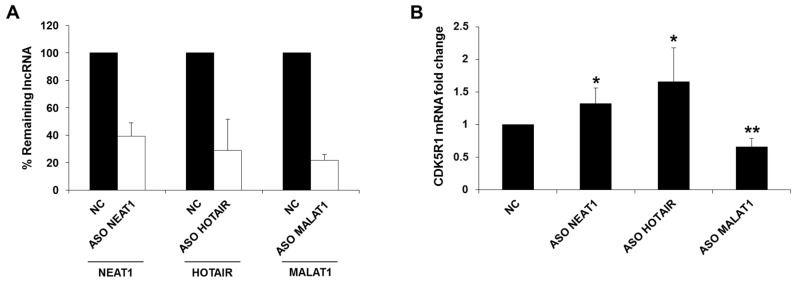
Effect of NEAT1, HOTAIR, and MALAT1 silencing on *CDK5R1* mRNA levels. (**A**) NEAT1, HOTAIR, and MALAT1 levels 24 h after transfection with specific ASOs. The levels of each lncRNA were reduced by at least 60%, compared to a control ASO (NC)-transfected cells. (**B**) Increased *CDK5R1* transcript levels were observed after NEAT1 and HOTAIR silencing, compared to the normal control. On the contrary, *CDK5R1* mRNA levels were significantly decreased after MALAT1 silencing. *n* = 5, mean ± s.d., * *p* < 0.05, ** *p* < 0.01, Student’s *t*-test.

In order to test the hypothesis that lncRNAs might be involved in the regulation of *CDK5R1*, we analyzed the effect of NEAT1, HOTAIR, and MALAT1 downregulation on *CDK5R1* expression. We transfected HeLa cells with 10 nM of specific 2’OMe-PS antisense oligonucleotides (ASO) to specifically knockdown the three lncRNAs. Total RNA was extracted 24 h after transfection and the levels of lncRNAs and *CDK5R1* mRNA were assessed by qRT-PCR. The analysis showed that NEAT1, HOTAIR, and MALAT1 levels were reduced by 61%, 71%, and 78% respectively, compared to the control oligonucleotide (Figure 1A). Remarkably, increased *CDK5R1* transcript levels were observed after NEAT1 and HOTAIR silencing, meaning that these two lncRNAs negatively regulate *CDK5R1* expression (Figure 1B). On the contrary, *CDK5R1* mRNA levels were significantly decreased after MALAT1 silencing compared to controls, indicating a positive action of this lncRNA on *CDK5R1* expression (Figure 1B).

### 2.2. NEAT1, HOTAIR, and MALAT1 Upregulate miR-15/107 Expression

**Figure 2:**
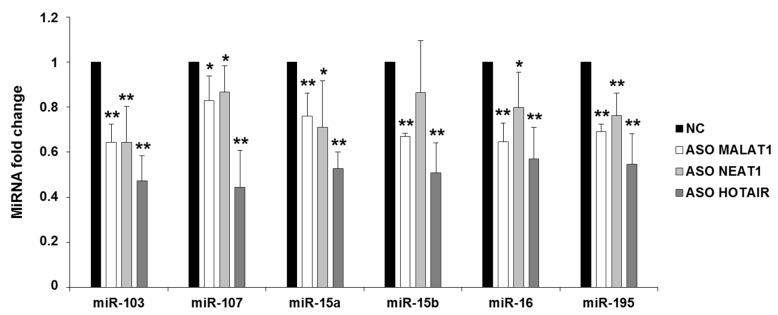
Effect of NEAT1, HOTAIR, and MALAT1 silencing on miR-15/107 levels. Decreased levels of all miR-15/107 miRNAs were detected after the knock-down of the three lncRNAs. *n* = 5, mean ± s.d., * *p* < 0.05, ** *p* < 0.005, Student’s *t*-test.

Since we previously demonstrated that *CDK5R1* expression is negatively regulated by the miR-15/107 group of microRNAs [11], we also verified by qRT-PCR on the RNA previously extracted from HeLa cells if NEAT1, HOTAIR, and MALAT1 silencing was able to affect miR-15/107 expression. The levels of all the analyzed miR-15/107 family members were reduced after NEAT1, HOTAIR, and MALAT1 silencing, compared to the control treatment (Figure 2), being HOTAIR the most efficient with a reduction of miRNA targets of about 50%. NEAT1 and MALAT1 led to a less pronounced but significant reduction of all miRNAs, with the exception of miR-15b after NEAT1 knock-down, whose reduction did not reach the statistical significance (Figure 2).

These data suggest that HOTAIR and NEAT1 might negatively regulate *CDK5R1* expression through a positive action on miR-15/107 levels. On the contrary, the positive effect of MALAT1 on *CDK5R1* mRNA cannot be explained by the action of these miRNAs, and a different mechanism must be involved in MALAT1-mediated positive effect on *CDK5R1* expression.

**Figure 3:**
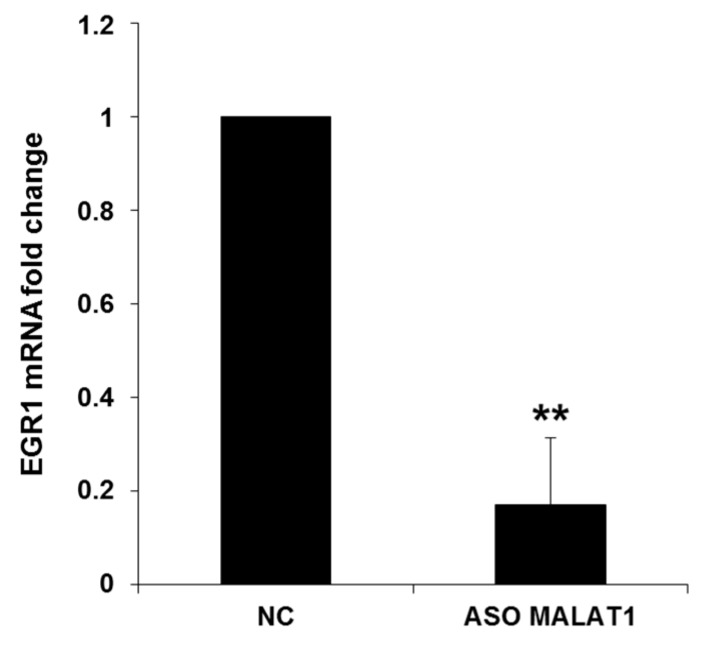
Effect of MALAT1 silencing on *EGR1* mRNA levels. *EGR1* mRNA levels were significantly decreased after MALAT1 silencing, compared to the normal control (NC). *n* = 3, mean ± s.d., ** *p* < 0.005, Student’s *t*-test.

*CDK5R1* also represents a target of EGR1 transcription factor whose expression is induced by ERK/MAPK pathway activation [30]. Since MALAT1 has been described to be a positive modulator of ERK/MAPK pathway [31], we evaluated the expression of *EGR1* following MALAT1 silencing. Consistently, qRT-PCR analysis showed that the levels of *EGR1* are strongly reduced (83%) in cells treated with MALAT1 specific antisense oligonucleotide, compared to normal control (Figure 3). These results suggest that the positive regulation exerted by MALAT1 on *CDK5R1* expression can be due to MALAT1-mediated enhancement of *EGR1* levels, likely overcoming the concurrent downregulation of miR-15/107 miRNAs.

### 2.3. NEAT1 is Upregulated in AD Temporal Cortex and Hippocampus

We recently showed that miR-15/107 miRNAs level is reduced in the hippocampus and the temporal cortex, but not in the cerebellum, of AD brains. Furthermore, we showed that increased *CDK5R1* mRNA levels are displayed by AD hippocampus tissue, compared to controls [11]. These data are consistent with the hypothesis that an increase of *CDK5R1* expression, and consequent enhanced CDK5 activity, caused by downregulation of the miR-15/107 family has a role in the pathogenesis of AD.

To verify whether NEAT1, HOTAIR, and MALAT1 expression is also altered in Alzheimer’s disease, we quantified their levels by qRT-PCR in the temporal cortex, hippocampus, and cerebellum of the same AD patients and age-matched healthy controls which were analyzed in our previous work.

**Figure 4:**
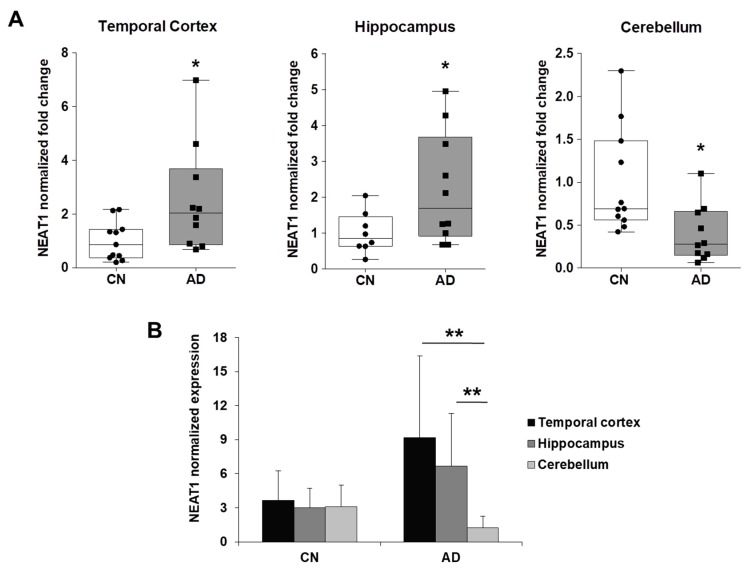
Comparison between the levels of NEAT1 expression in AD and control brain tissues. (**A**) Dot-Box-plots of the levels of NEAT1 expression in three different brain areas (temporal cortex, hippocampus, and cerebellum) of AD patients (*n* = 10) and controls (*n* = 8–11). Dark horizontal lines represent the median, with the box representing the 25th and 75th percentiles, the whiskers the 5th and 95th percentiles. The average of control values was set to 1 and all values were calculated relatively. NEAT1 levels are significantly upregulated in temporal cortex and hippocampus and downregulated in cerebellum of AD patients, compared to control individuals. (**B**) Higher NEAT1 expression levels were observed in temporal cortex and hippocampus, compared to cerebellum, in AD patients, but not in control individuals. * *p* < 0.05, ** *p* < 0.01, Student’s *t*-test.

**Figure 5:**
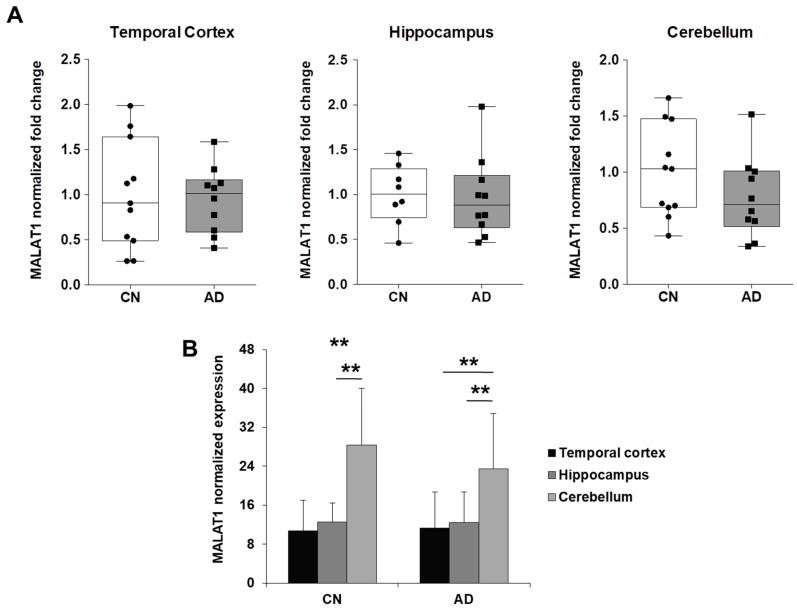
Comparison between the levels of MALAT1 expression in AD and control brain tissues. (**A**) Dot-Box-plots of the levels of MALAT1 expression in three different brain areas (temporal cortex, hippocampus, and cerebellum) of AD patients (*n* = 10) and controls (*n* = 8–11). Dark horizontal lines represent the median, with the box representing the 25th and 75th percentiles, the whiskers the 5th and 95th percentiles. The average of control values was set to 1 and all values were calculated relatively. We observed no difference in MALAT1 levels between AD patients and control individuals in any analyzed tissues. (**B**) Higher MALAT1 expression levels were observed in cerebellum, compared to temporal cortex and hippocampus, in both AD patients and controls individuals. ** *p* < 0.01, Student’s *t*-test.

Remarkably, we found that NEAT1 was significantly overexpressed in temporal cortex and hippocampus and downregulated in cerebellum of AD patients, compared to control individuals (Figure 4A). Comparing NEAT1 distribution among the different brain areas of control individuals we observed similar expression levels, while NEAT1 was significantly higher in temporal cortex and hippocampus compared to cerebellum in AD patients (Figure 4B). On the contrary, MALAT1 expression showed no difference between AD patients and controls (Figure 5A), even though higher levels were detected in cerebellum, compared to temporal cortex and hippocampus, in both groups (Figure 5B). Finally, HOTAIR was expressed at very low levels in hippocampus and cerebellum and was not detectable in temporal cortex. Particularly, HOTAIR was downregulated in cerebellum in AD patients, compared to controls. No difference in HOTAIR expression between hippocampus and cerebellum was observed in both groups.

### 2.4. NEAT1 and CDK5R1 Overexpression as a Biomarker of AD

In order to verify the existence of a correlation between NEAT1 and *CDK5R1* expression in AD and control brain tissues, we performed a Pearson’s correlation analysis between the normalized expression levels of NEAT1 and those previously obtained for *CDK5R1* [11].

**Figure 6:**
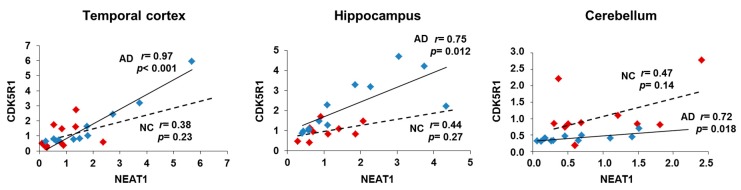
Correlation analysis between NEAT1 and *CDK5R1* expression in temporal cortex, hippocampus and cerebellum samples of AD patients (blue diamonds) and controls (red diamonds). *r* = Pearson's correlation coefficient, solid line = linear regression line of AD patients, dashed line = linear regression line of normal controls (NC).

The analysis showed that a significant positive correlation between *CDK5R1* and NEAT1 levels was only displayed by AD patients’ postmortem specimens of hippocampi and temporal cortices with very significant values of Pearson’s *r* (Figure 6). In other words, in AD, NEAT1 increases along with *CDK5R1*, indicating a peculiar functional relationship (in vitro defined as a negative NEAT1 control over *CDK5R1*) which is specific for AD and that can be either a protective response-related mechanism aimed at limiting (inefficiently) *CDK5R1* upregulation or part of the disease pathogenesis. Notably, in the cerebellum, a brain area that is almost unaffected by the disease and in which *CDK5R1* does not appear to be upregulated, the correlation between NEAT1 levels and those of *CDK5R1* is still evident only in AD. This observation suggests that in the cerebellum a protective, NEAT1-associated mechanism might efficiently control *CDK5R1* levels.

**Figure 7:**
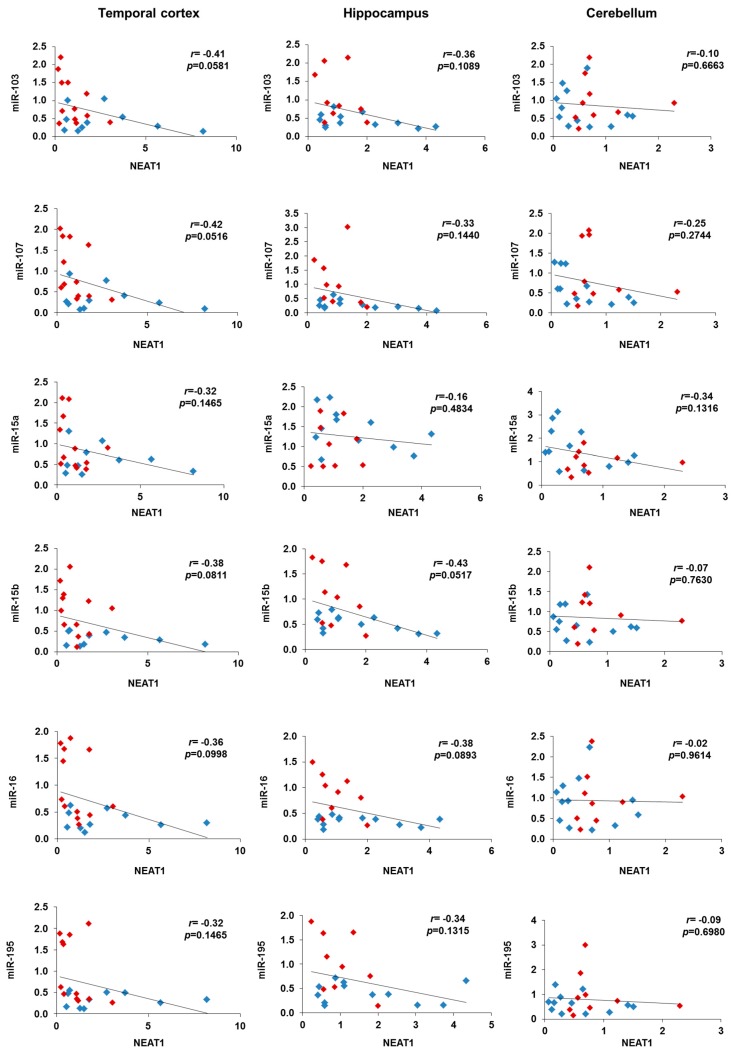
Correlation analysis between miR-103, miR-107, miR-15a, miR-15b, miR-16, and miR-195 and NEAT1 expression in temporal cortex, hippocampus, and cerebellum samples of AD patients (blue diamonds) and controls (red diamonds). *r* = Pearson’s correlation coefficient.

Another interesting observation is the opposite correlation between the expression of NEAT1 and miR-15/107 miRNAs in AD brains and controls. Indeed, while in controls we observed high expression of miR-15/107 and low expression of NEAT1, in AD patients, to higher NEAT1 levels correspond very low miR-15/107 levels, particularly in temporal cortex and hippocampus (Figure 7). In conclusion, a picture emerges in which not only NEAT1 is unable to increase its levels in a sufficient manner to counteract *CDK5R1* increase in AD brains, but also, it loses the ability to positively regulate miR-15/107, validated negative regulators of *CDK5R1*. In this way, converging pathological mechanisms based on a failure of lncRNA NEAT1 and miR-15/107 homeostatic role towards *CDK5R1* expression could result in *CDK5R1* upregulation.

## 3. Discussion

Cyclin-dependent kinase 5 (CDK5) has a major role in CNS development and functioning and its deregulation can contribute to different pathological events implicated in the pathogenesis of Alzheimer’s disease [8]. Monomeric CDK5 itself does not display kinase activity and requires, in order to be active, the association with its regulatory subunits, p35 or p39, although p35, encoded by the *CDK5R1* gene, is considered the most important CDK5 activator [32]. Multiple layers of regulation govern *CDK5R1* expression and ensure p35 levels and CDK5 activity to be tightly controlled. They include transcriptional activation by EGR1 transcription factor and repression by LSD1 demethylase [29,30], as well as well various post-transcriptional mechanisms which involve the binding to the long and evolutionary conserved *CDK5R1* 3′-UTR of both RNA-binding proteins and microRNAs [9,10,11].

In this work, we took into account another class of non-coding RNAs, long non-coding RNAs (lncRNAs), as potential regulators of *CDK5R1* expression. In particular, our attention was focused on three lncRNAs, NEAT1, HOTAIR, and MALAT1. Our results showed that these three lncRNAs are able to influence *CDK5R1* expression. In particular, NEAT1 and HOTAIR exert a negative regulatory effect on *CDK5R1* levels, while MALAT1 has an opposite, positive action. In addition, all these lncRNAs were proven to positively regulate the miRNAs belonging to the miR-15/107 family.

We hypothesize that the negative regulatory effect of NEAT1 on *CDK5R1* expression might depend on its capacity to exert a positive control on miR-15/107 levels. Interestingly, we also found that NEAT1 is significantly overexpressed in temporal cortex and hippocampus of AD patients, compared to control individuals, suggesting that NEAT1 upregulation can be considered a biomarker of the disease. Recent studies have linked altered expression and function of long non-coding RNAs to the pathogenesis of neurodegenerative diseases (reviewed in [22]). In particular, different lncRNAs have been found to be dysregulated in Alzheimer’s disease (e.g., BACE1-AS and NDM29) and to be involved in AD pathogenesis by promoting β-amyloid production. In this work we show that, in vitro, NEAT1 negatively regulates *CDK5R1* expression. In line, NEAT1 upregulation in AD patients would predict a corresponding downregulation of *CDK5R1*. Notably, this was not the case. Indeed, in AD brains the expression of both *CDK5R1* and NEAT1 is increased compared to healthy controls. A possibility is that the negative control of NEAT1 over *CDK5R1* levels is not efficient either because the ratio between *CDK5R1* and NEAT1 typical of controls is increased in AD brains (Figure 6), or because NEAT1 loses its positive control towards miR-15/107 (Figure 7). As a result, we can infer that the critical NEAT1 level that would be necessary to counteract *CDK5R1* expression is not reached in AD temporal cortex and hippocampus. For these reasons, NEAT1 overexpression as a pathomechanism in Alzheimer’s disease is unlikely, although our data do not allow to fully reject this hypothesis. Moreover, several lines of evidence suggest that NEAT1 and paraspeckles may have a neuroprotective role in neurodegenerative diseases. An increase in paraspeckles formation and NEAT1 levels has been detected in spinal motor neurons of early phase amyotrophic lateral sclerosis (ALS) patients compared to control individuals [33] and compromised paraspeckles formation has been proposed as a pathogenic factor in FUSopathies [34]. Moreover, NEAT1 levels are also increased in the brains of patients affected by frontotemporal lobar degeneration (FTLD) [35]. Importantly, Sunwoo and colleagues [36] showed that NEAT1 is overexpressed in Huntington’s disease patients and plays a protective role against cell injury. These data suggest that NEAT1 may contribute to neuronal survival in the degenerating brain. Analogously, our work showed that NEAT1 is also overexpressed in AD patients. In this context, putative beneficial effects of NEAT1 are still unknown. However, enhanced amounts of *CDK5R1* are predicted to cause CDK5 hyperactivation, which is a typical hallmark of the disease [8]. It is worth noting that CDK5 can phosphorylate p53, which is also known to be upregulated in AD [37], thereby inducing its stabilization and transcriptional activation, contributing to neuronal cell death [38]. Remarkably, p53 was recently demonstrated to activate NEAT1 expression [39]. These findings provide a possible molecular link between *CDK5R1* and NEAT1 upregulation in AD brains, albeit they do not indicate the reason why *CDK5R1* escapes NEAT1 control in AD condition.

The negative action exerted by HOTAIR on *CDK5R1* expression is likely mediated by different converging mechanisms. On the one hand, HOTAIR can negatively regulate *CDK5R1* at the post-transcriptional level via the same miR-15/107 miRNA-mediated mechanism as NEAT1, on the other hand it could regulate *CDK5R1* also at the transcriptional level participating to recruiting and regulating the LSD1 and PRC2 repressing complexes [28]. Interestingly, HOTAIR also represses the transcription of *BDNF* [27], which normally induces the ERK-mediated expression of *CDK5R1* [29].

On the contrary, our silencing experiments suggest that MALAT1 positively affect *CDK5R1* expression. Since reduction of miR-15/107 levels after MALAT1 silencing would predict an increase in the amount of *CDK5R1* mRNA, as expected for their inhibitory action, there must be other predominant regulatory mechanisms leading to *CDK5R1* upregulation by MALAT1. As we have also shown that MALAT1 silencing causes a strong reduction in the levels of EGR1, which is the main activator of *CDK5R1* transcription, we thus speculate that MALAT1 can enhance *CDK5R1* expression mainly by upregulating EGR1 transcription factor through activation of ERK/MAPK signaling pathway [31].

Moreover, the activation of this pathway is known to play a critical role in promoting neurite outgrowth [31]. Given that the p35/CDK5 complex is also essential for neurite outgrowth during neuronal differentiation [40], our evidence raises the interesting hypothesis that MALAT1 induces axonal elongation via *CDK5R1*/p35 upregulation.

Mounting evidence suggests that lncRNAs can function as miRNA sponges, by sequestering the mature miRNA molecules and preventing the binding to their target mRNAs [41]. However, this mechanism is predicted to increase—or leave unchanged—the levels of the sequestered miRNAs when the lncRNA acting as sponge is silenced [14,42]. Since we observed that the silencing of NEAT1, HOTAIR, and MALAT1 lead to a reduction of miR-15/107 miRNAs, we hypothesize that this effect could be mediated by a positive regulatory action of these lncRNAs on transcription factors that promote the expression of this family of miRNAs or, alternatively, by their interaction with the microprocessor to enhance pri-miRNA processing, as already demonstrated for NEAT1 [43].

Overall, our data suggest that lncRNAs can provide a further layer and a higher degree of complexity to the control of *CDK5R1* expression. In addition, we show that NEAT1 is upregulated in AD brain, possibly as a part of a protective mechanism against neuronal death, and can be considered a marker of the disease and represents a potential pharmacological target for therapeutic intervention in AD.

## 4. Materials and Methods 

### 4.1. Cell Cultures

HeLa cells (code CCL-2, ATCC, Manassas, VA, USA) were cultured in DMEM high glucose (Euroclone, Pero, Italy) medium with 10% fetal bovine serum (FBS) (Euroclone), 100 U/mL penicillin-streptomycin (Euroclone) and 0.01 mM l-glutamine (Euroclone). Cultures were maintained at 37 °C in a 5% CO_2_ incubator.

### 4.2. Brain Tissues

Post-mortem frozen brain tissue samples of AD patients and age- and sex-matched non-demented individuals were obtained from MRC London Neurodegenerative Diseases Brain Bank (King’s College London), Newcastle Brain Tissue Resource (Newcastle University), and South West Dementia Brain Bank (University of Bristol) and are described in [10]. The approval of the Ethics Committee of the University of Milan was obtained for the use of post-mortem tissues for research purposes (Project identification code: RV_RIC_AT16MVENT_M, 15 June 2016).

### 4.3. Antisense Oligonucleotides Transfection

2′-*O*-methyl phosphorothioate antisense oligonucleotides (2′OMe-PS ASO) were designed as described by [44] (NEAT1 1473, HOTAIR 1259, MALAT1 5326, NC1) and purchased from Consorzio Futuro in Ricerca, Università degli Studi di Ferrara (Ferrara, Italy).

ASOs were used at 10 nM concentration. 200 × 10^3^ HeLa cells were seeded in 6-well plates in order to extract total RNA. The cells were transfected 24 h after seeding with 2′OMe-PS ASOs, using Lipofectamine 2000 (Thermo Fisher Scientific, Waltham, MA, USA) transfection reagent according to the manufacturer’s instructions. Cell extracts were prepared for analysis 24 h after the transfection.

### 4.4. Real-Time PCR

Total RNA from transfected/nontransfected cells and from brain tissues (100 mg of each sample tissue) was isolated using TRIzol reagent (Thermo Fisher Scientific), according to the manufacturer’s instructions. Concentration and purity of RNA were measured using the Nanodrop spectrophotometer (ThermoFisher Scientific). All RNA samples had an A260/280 value of 1.8–2.1.

For the measurement of *CDK5R1*, *EGR1* mRNA, NEAT1, HOTAIR, and MALAT1 RNA, a DNase reaction was performed on 1 μg of total RNA using RQ1 RNase-Free Dnase (Promega, Madison, WI, USA) and then cDNA was synthetized in 20 μL reactions using the High Capacity cDNA Reverse Transcription Kit (Thermo Fisher Scientific), according to the manufacturer’s instructions. SYBR Green Real-Time PCR was performed using the GoTaq qPCR Master Mix (Promega) and the following primers: *CDK5R1* fw: TGAGCGGGTCTAGTGGAAAG; *CDK5R1* rev: AGCAGCAGACAAGGGGGTAG; *EGR1* fw: GAGCACCTGACCGCAGAGTC; *EGR1* rev: GTGTTGCCACTGTTGGGTGC; HOTAIR fw: GGCAAGACGGGCACTCACAG; HOTAIR rev: CTGGGCGTTCATGTGGCGAG; MALAT1 fw: AGGGAAAGCGAGTGGTTGGT; MALAT1 rev: GAAATCGGCCTACGTCCCCA; NEAT1 fw: CGGAGGTGAGGGGTGGTCTG; NEAT1 rev: GCAGTCCCCGCCTGTCAAAC; *EIF4A2* fw: GGTCAGGGTCAAGTCGTGTT; *EIF4A2* rev: CCCCCTCTGCCAATTCTGTG; *CYC1* fw: TAGAGTTTGACGATGGCACCC; *CYC1* rev: CGTTTTCGATGGTCGTGCTC; *SYP* fw: CTTCGCCATCTTCGCCTTTG; *SYP* rev: TACACTTGGTGCAGCCTGAAG; *ENO2* fw: CTGAAGCCATCCAAGCGTGC; *ENO2* rev: CCCACCACCAGGTCAGCAAT. 20 μL PCR reactions were prepared with 2× SYBR Green mix containing 1.6 μL of reverse transcriptase product and 0.4 μL of each primer (10 µM). The PCR mixtures were incubated at 95 °C for 3 min, followed by 40 cycles of 95 °C for 10 s, 60 °C for 20 s, and 72 °C for 10 s. The calculation of gene expression levels was based on the ΔΔ*C*t method in transfection experiments and on the Δ*C*t method for gene expression analysis in brain tissues. The geometric mean of the expression values of *EIF4A2* and *CYC1* housekeeping genes was used as internal control in transfection experiments, while gene expression levels in brain tissues were normalized on the geometric mean of the same housekeeping genes and the neuronal markers *SYP* and *ENO2*.

For the measurement of miRNAs, a two-step Taq-Man real-time PCR assay was performed using primers and probes obtained from Thermo Fisher Scientific. The reverse transcriptase reaction was performed using the TaqMan MicroRNA Reverse Transcription kit (Thermo Fisher Scientific), according to the manufacturer’s instructions. cDNA was synthesized from 50 ng of total RNA in 15 μL reactions, using the stem-loop primer for miR-15a (ID000389), miR-15b (ID000390), miR-16 (ID000391), miR-103 (ID000439), miR-107 (ID000443), miR-195 (ID000494), and U6 snRNA (ID001973). The PCR reaction (20 μL) contained 1.3 μL of reverse transcriptase product, 10 μL of Taq-Man Universal PCR Master Mix (Thermo Fisher Scientific) and 1 μL of the appropriate TaqMan MicroRNA Assay (20×) containing primers and probes for the miR of interest. The PCR mixtures were incubated at 95 °C for 10 min, followed by 40 cycles of 95 °C for 15 s and 60 °C for 60 s. The expression of miRs was based on the ΔΔ*C*t methods, using U6 snRNA as an endogenous control. All PCRs were performed in triplicate using an iQ5 Real-Time PCR detection system (Bio-Rad, Hercules, CA, USA).

### 4.5. Statistical Analysis

Each experiment was carried out at least three times. Histograms represent the mean values and bars indicate the standard deviation of the mean. The box plots show median, 25th and 75th percentile values and whiskers to the minimum and maximum value. The statistical significance of the results was determined using Student’s *t*-test, with data considered significant when *p* < 0.05. The degree of linear relationship between *CDK5R1* gene and NEAT1 expression levels was calculated using Pearson’s correlation coefficient (*r* value). The *p* value was calculated from an extra sum-of-squares *F* test.
